# Spontaneous hemothorax in 4 COVID-19 ARDS patients on VV-ECMO revealing pulmonary artery aneurysms

**DOI:** 10.1186/s13054-020-03359-7

**Published:** 2020-11-06

**Authors:** Cyrielle Desnos, Samia Boussouar, Guillaume Hekimian, Alban Redheuil, Alain Combes

**Affiliations:** 1 Service de Médecine Intensive Réanimation, Hôpital La Pitié-Salpêtrière, Assistance Publique-Hôpitaux de Paris (AP-HP), Sorbonne Université, Paris, France; 2 LIB-Laboratoire D’imagerie Biomédicale, INSERM, CNRS, ICAN Institute of CardioMetabolism and Nutrition, ACTION Study Group, Cardiothoracic Imaging Unit, Hôpital Pitié-Salpêtrière (AP-HP), Sorbonne Université, Paris, France; 3 Institut de Cardiométabolisme et Nutrition (ICAN), Hôpital La Pitié-Salpêtrière, Assistance Publique-Hôpitaux de Paris (AP-HP), Sorbonne Université, Paris, France

COVID-19 pneumonia is a cause of severe ARDS. Its pathophysiology involves endothelial dysfunction and angiogenesis related to ACE-2 receptor, the host-cell receptor for SARS-CoV-2, expressed by endothelial cells, which may lead to thrombosis or hemorrhage [1].

This case series describes the presentation of COVID-19 patients who had unusual spontaneous hemothorax while on veno-venous extra corporeal membrane oxygenation (VV-ECMO) for severe ARDS. In accordance with French legislation, only non-opposition of patient’s surrogate for utilization of the deidentified data was obtained. The ICU database was registered with the national data protection authority (CNIL 1950673). From February to September 2020, 62 patients with confirmed COVID-19-related severe ARDS requiring VV-ECMO were transferred and treated in our tertiary care ICU, of whom 4 had spontaneous hemothorax.

Ages were 33, 63, 48 and 46 years; 2 patients were women. None of them had pulmonary embolism on previous CT angiography, and they had received continuous infusion of unfractionated heparin at high preventive dose according to international expert guidelines [2]. They were on VV-ECMO for 3, 9, 22 and 28 days at the time of the hemothorax onset. In all four patients, hemothorax was revealed by shock, both hemorrhagic and obstructive by compression of the mediastinum, requiring high doses of catecholamine and massive packed red blood cells transfusion. One patient experienced cardiac arrest shortly after shock onset, resuscitated after chest drainage. On chest CT scan, vascular lung abnormalities were very similar in the 4 patients, including peripheral medium and small pulmonary artery branches aneurysms, one of them also having renal and diaphragmatic artery aneurysms (Fig. 1). Three of the patients underwent salvage radioembolization, one of them survived to this episode without relapse, and hemothorax was fatal for the 3 others.

**Fig. 1:**
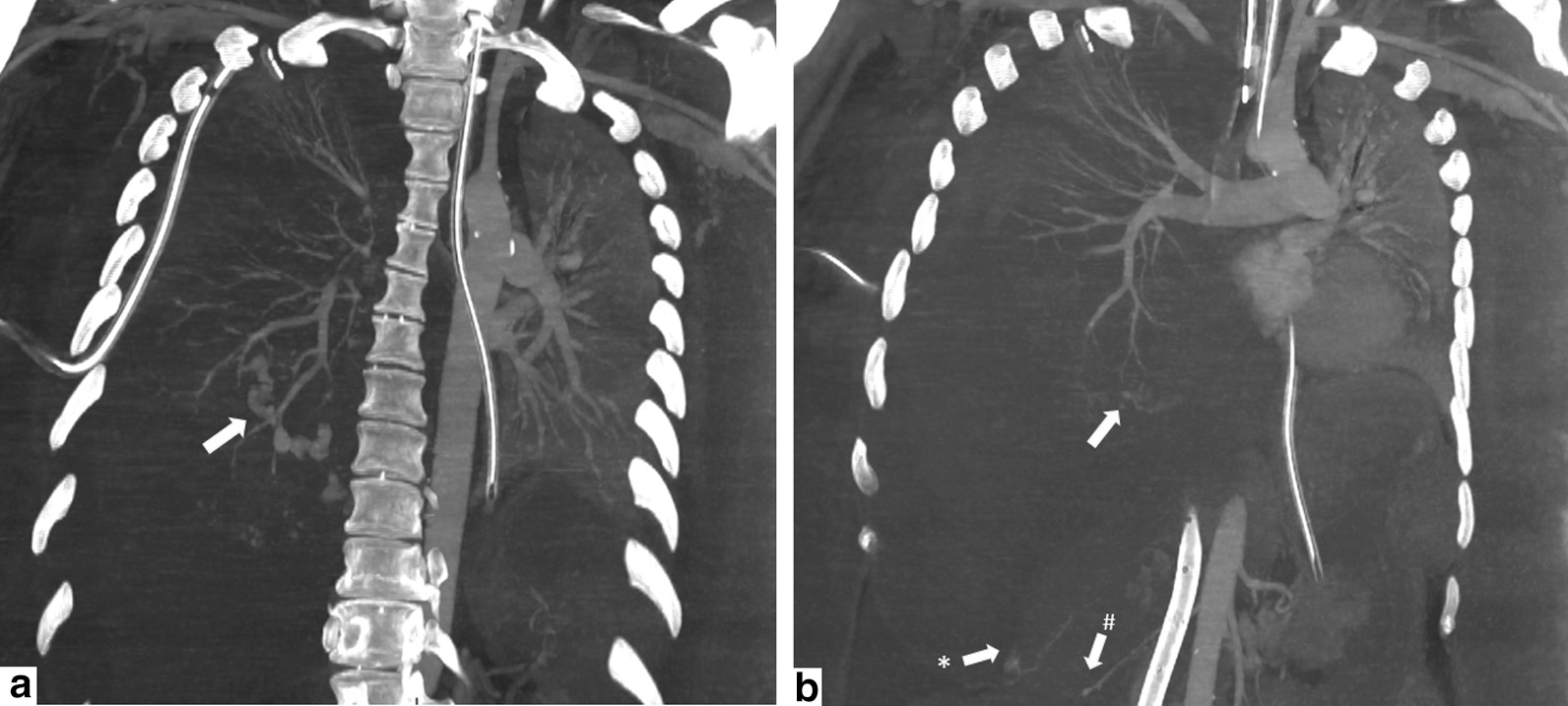
Coronal Maximum Intensity Projection (42 mm in **a** and 8 mm in **b**) chest CT images of a 46-year-old male with COVID-19 pneumonia. Note the presence of arterial pulmonary aneurysms complicated by intraparenchyma bleeding (**a** arrow), diaphragmatic (**b** star) and kidney aneurysms (**b** dash). An apical right pleural drain and gastric tube (panel **a**, **b**) as well as veno-venous ECMO superior and inferior canulae (panel **b**) can also be seen

Coagulation parameters a few hours before the diagnosis of hemothorax were within normal ranges: Fibrinogen ranged between 2.4 and 6.8 g/l, platelet count between 76 and 196 G/l, prothrombin time between 12 and 14 s, activated partial thromboplastin time (aPPT) between 32 and 55 s, and anti-Xa activity was < 0.22 IU/ml in all 4 patients.

To our knowledge, this is the first series of hemothorax related to vascular abnormalities complicating severe ARDS of any cause during ECMO run.

These patients experienced severe forms of COVID-19, including severe multisystem inflammatory state [3]. This commonly called “cytokine storm” is associated with a pro-coagulant state, whereas extracorporeal life support with ECMO is known to cause hemostasis and coagulation disorders potentially promoting bleeding [4] and therefore hemothorax.

The aneurysms observed on the CT scans of our four patients, developed in a highly inflammatory setting in patients with positive SARS-CoV2 viremia. Children are also concerned by vasculitis-like COVID-19 forms with multisystem inflammatory syndrome and cardiac involvement, including, in less than 20% of cases, coronary artery aneurysm [5]. The CT scan aspect of the pulmonary artery aneurysms observed in our patients was close to those observed in polyarteritis nodosa [6], suggesting a vasculitis-like pathophysiological mechanism in these critically ill COVID-19 patients. Physicians and radiologists should be aware of the occurrence and potential fatal outcomes of such vascular abnormalities which should be systematically searched in patients with the most severe forms of COVID-19.

## Data Availability

Not applicable.
